# Antibiotic usage in a South African paediatric medical ward following the introduction of an antibiotic prescription chart

**DOI:** 10.11604/pamj.2023.45.26.36548

**Published:** 2023-05-08

**Authors:** Habibat Dolapo Salau, Ané Orchard, Sarah Stacey, Sheeba Varughese, Deanne Johnston, Razeeya Khan

**Affiliations:** 1 Department of Pharmacy and Pharmacology, University of the Witwatersrand, Johannesburg, South Africa,; 2 Department of Internal Medicine, University of the Witwatersrand, Johannesburg, South Africa,; 3 Department of Paediatric and Child Health, University of the Witwatersrand, Johannesburg, South Africa,; 4 Discipline of Pharmaceutical Sciences, University of KwaZulu Natal, Johannesburg, South Africa

**Keywords:** Antibiotic use, antibiotic resistance, antimicrobial stewardship, paediatric

## Abstract

**Introduction:**

the global rise in antibiotic resistance (ABR), coupled with a dry pipeline for the discovery of new antibiotics requires the conservation of currently available antibiotics. Antimicrobial stewardship (AMS) interventions are being implemented to optimize antibiotic use including the use of antibiotic prescription charts. This study reviewed the use of antibiotics before and after the introduction of an antibiotic prescription chart in a paediatric medical ward of an academic tertiary hospital in Johannesburg.

**Methods:**

a cross-sectional retrospective review of patient records was conducted for patients admitted to a paediatric medical ward of an academic tertiary hospital over two study periods; before and after the introduction of an antibiotic prescription chart. Data were captured on a Microsoft® Excel (2010) spreadsheet and analyzed using Stata/IC 15.1 (StataCorp, USA).

**Results:**

antibiotic use decreased significantly by 7.04% following the introduction of the antibiotic prescription chart (p=0.027). Fields often left unfilled on the antibiotic prescription chart include age (100%), a record of renal function (GFR/CrCl) (97.46%), time of antibiotic prescribing (83.62%) and a record of culture and sensitivity results (80.17%).

**Conclusion:**

the findings of this study show an improvement in antibiotic use, the frequency of culture and sensitivity testing and documentation of relevant parameters after the introduction of the antibiotic prescription chart. The use of an antibiotic prescription chart is a practical way to achieve optimal antibiotic use and to encourage proper detailing of the clinical components necessary for antibiotic selection in a hospital setting in a developing country.

## Introduction

The unabating challenge of the development of bacterial resistance coupled with a dry antimicrobial drug pipeline has made antibiotic resistance a global public health challenge [1]. Antibiotics are the most commonly used medicines worldwide with usage rates as high as 50% in hospitalized patients [2,3]. A global “post-antibiotic era” is approaching where available antibiotics will lose their benefits [4,5]. This places a significant social and financial burden on society, with increased cost and prolonged hospital stays, leading to increased morbidity and mortality [1,6]. The severity of the antimicrobial resistance (AMR) crisis in developing countries, including South Africa, is weighty and worsened by the limited availability and affordability of antimicrobials essential in the treatment of resistant organisms [7]. The first reported case of carbapenemase-producing bacteria in Africa emerged in South Africa in 2011 [8]. Surveillance data suggest that resistance continues to rise in prevalent infectious pathogens [6]. Antibiotic usage, whether indicated or not, as well as improper antibiotic prescribing practices, are significant contributors to the emergence of resistant bacteria [9,10]. Infection prevention and control (IPC) programs, alongside antimicrobial stewardship strategies, are among the approaches in the fight against antibiotic resistance worldwide [11]. Antimicrobial stewardship (AMS) was endorsed at the sixty-eighth World Health Assembly of the World Health Organization (WHO) as a global strategy aimed at reducing AMR [12]. It was recommended that all member states adopt individual national action plans to combat AMR. The objective of AMS is to encourage the responsible use of antibiotics to improve patient outcomes [13]. Antimicrobial stewardship is crucial to combat antibiotic resistance [14] to reduce the advent of resistant microbes, unnecessary drug use, adverse effects, and costs [15-17]. One of the strategic objectives of antimicrobial stewardship is the implementation of an antibiotic prescription chart to promote the rational use of antimicrobials by facilitating surveillance and improving compliance and quality of prescribing [18,19]. A weekly AMS ward round in addition to a customized antibiotic prescribing chart introduced in two medical wards of a South African academic teaching hospital led to a 19.6% reduction in antibiotic usage, without compromising patient care [20]. However, it was difficult to isolate the individual impact of the chart from that of the ward rounds [20]. The antibiotic chart design includes information relating to patient demographics (name, age, weight, ward, gender, and renal function), diagnosis, infection source, antibiotics (antibiotic, dosage, route of administration, dosing frequency, time of prescribing and duration of therapy), the rationale for antibiotic prescribing, as well as culture and sensitivity testing (timing of culture testing, isolated pathogen, susceptible and resistant antibiotics). An antibiotic prescription chart is important in all stages of the antibiotic prescribing process from patient evaluation to dispensing of antibiotics. It also serves as a crucial data recording tool, allowing members of the AMS and healthcare team to access essential information throughout the duration of antibiotic therapy. Studies of AMS practices and the impact of introducing an antibiotic prescription chart in South Africa are scarce. Hence, this study aimed to investigate the utilization of antibiotics after introducing a paper-based antibiotic prescription chart in an academic tertiary hospital in Johannesburg.

## Methods

**Setting:** this study was conducted in a paediatric medical ward of a public academic tertiary hospital in Johannesburg, South Africa. The hospital has 1088 beds with over 4,000 staff members and functions as a referral hospital for several hospitals in its cluster. The paediatric medical ward consists of 24 beds and admits patients under the age of six with medical and non-infectious diagnoses.

**Study design:** a retrospective record review of antibiotic usage was carried out across two study periods. The first study period was between 1^st^ August and 31^st^ October 2016, which represents the period before the introduction of the antibiotic prescription chart (pre-introduction), while the second study period was between 1^st^ August and 31^st^ October 2019 and represents the period after the introduction of the antibiotic prescription chart (post-introduction).

**Data collection and statistical analysis:** records of all patients (male and female) admitted to the study ward, who received an antibiotic throughout the study periods, were included in the study. Data retrieved included patient demographics (age, weight, gender), diagnosis, source of infection, prescribed antibiotics, antibiotic dosage, frequency of dosing, route of administration as well as microbial culture and sensitivity testing. Data were retrieved and analyzed using Stata/IC 15.1 (StataCorp, USA). Patient characteristics and all outcome measures were quantitatively compared for both study periods and presented using descriptive analysis and comparison tables. Pearson´s Chi-square was used to test associations between selected categorical variables. A p-value not exceeding 0.05 was regarded as significant.

**Data reliability and validity:** data were collected by the same researcher. Unique study numbers were assigned to each patient record for easy tracing. The same variables were collected throughout the study and sorted before data analysis. Other studies have used a similar data collection tool [4,20]. The same months across both study periods were used to mitigate the possibility of changes in disease patterns due to changes in season.

**Ethical considerations:** ethical clearance to conduct this study was obtained from the University of the Witwatersrand Human Research Ethics Committee (ref. no. M190711).

## Results

A total of 336 patient records were reviewed with 225 patients receiving an antibiotic: 109 in the pre-introduction period and 116 in the post-introduction period.

**Antibiotic utilization:** this study found that antibiotics were prescribed to 70.78% of patients in the pre-introduction period and 63.74% in the post-introduction period, with a statistically significant difference in antibiotic prescribing across both study periods (p=0.027). The mean ± SD number of antibiotics prescribed per patient in the pre-introduction period was 1.91 ± 1.14 and 1.96 ± 1.08 in the post-introduction period with no statistically significant difference (p=0.3679). Three or fewer antibiotics per admission were frequently prescribed per patient over both study periods, representing 92.66% and 90.52% of patients in the pre-introduction and post-introduction periods respectively (Table 1).

**Table 1:** number of antibiotics prescribed per patient across both study periods

Number of antibiotics per patient	Pre-introduction (%)	Post-introduction (%)
1	45.87	40.52
2	31.19	36.21
3	15.60	13.79
4	3.67	6.03
≥ 5	3.67	3.45

**Microbial culture and sensitivity testing:** microbial culture and sensitivity tests were requested in 77.98% and 83.62% of patients in the pre-introduction and post-introduction periods respectively with no statistically significant difference (p = 0.710) in the number of tests requested across both study periods.

**Duration, antibiotics prescribed and routes of administration:** antibiotic regimens of one to seven days were frequently administered across both study periods (Table 2). On average, patients were treated with an antibiotic for 5.59 days in the pre-introduction period and 6.14 days in the post-introduction period. Antibiotics were commonly administered empirically across both study periods.

**Table 2:** diagnoses, duration of antibiotic therapy, commonly prescribed antibiotics and routes of administration across both study periods

		Pre-introduction (%)	Post-introduction (%)
**Common diagnoses**	Pneumonia	44.04	43.96
	Sepsis	10.09	27.59
	Lower respiratory tract infection	9.17	6.03
**Duration of antibiotic therapy**	1-7 days	82.57	81.04
	8-14 days	14.68	12.94
	15-20 days	1.84	3.44
	> 21 days	0.92	2.58
**Antibiotics commonly prescribed**	Ampicillin	39.45	36.21
	Gentamycin	29.36	26.72
	Cefotaxime	27.52	12.07
	Amoxicillin/clavulanic acid	22.94	30.17
**Common routes of administration**	Parenteral	58.72	65.52
	Oral and parenteral	29.35	18.10

**The use of the antibiotic prescription chart in the post-introduction period:** antibiotics were prescribed on 85.59% of antibiotic prescription charts in the post-introduction period (Table 3). Half (50%) of the antibiotic prescription charts did not have a source of infection recorded (Table 3). Furthermore, the timing of cultures sent (before or after antibiotic initiation) was only indicated on 47.41% of antibiotic prescription charts. The time of antibiotic prescribing was indicated on only 16.38% of antibiotic prescription charts (Table 3). The commonly omitted fields on the antibiotic prescription chart included age (100%), a record of renal function (GFR/CrCl) (97.46%), time of antibiotic prescribing (83.62%), a record of culture and sensitivity results (80.17%), the rationale for antibiotic prescribing (50%), a record of infection source (50%), and timing of culture tests (52.59%).

**Table 3:** audit of the antibiotic prescription chart in the post-introduction period

	Percentage of data entered on the antibiotic prescription chart (%)
**Patient demographics**	
Age	0.00
Weight	58.47
Allergies	43.22
Renal function	2.54
**Patient diagnosis**	78.81
**Infection source**	
Not recorded	50.00
Community	42.24
Hospital	6.90
Both	0.86
**Timing of culture**	
Before antibiotic	47.41
After antibiotic	0.00
Not documented	52.59
Culture and sensitivity results	19.83
Record of antibiotics	84.48
Time of antibiotic prescribing	16.38
The rationale for antibiotic prescribing	50.00

## Discussion

The introduction of an antibiotic prescription chart as an AMS strategy is beneficial in improving antibiotic use in healthcare institutions. The antibiotic prescription chart serves as a means of surveillance and enables the proper selection of antibiotics when used optimally. This study sought to review antibiotic use before and after implementing an antibiotic prescription chart in a paediatric medical ward of an academic tertiary hospital.

**Antibiotic utilization:** this study recorded a total of 70.78% and 63.74% of patients being administered at least one antibiotic in the pre-introduction and post-introduction periods respectively. Other studies have reported higher ranges of antibiotic prescribing of 72-84% [21-24] per patient. Antibiotic utilization in this study was, however, higher when compared to the WHO recommendation of below 30% utilization [25] and in studies conducted in other developing countries, including Ethiopia (52.3%) [26], Ghana (55.2%) [27] and Nigeria (51.0%) [28]. Inappropriate antibiotic use is a promoter of antimicrobial resistance, leading to increased treatment costs and prolonged hospital stay [1,6,29]. Furthermore, the proportion of antibiotic prescriptions was observed to have decreased significantly (p=0,027) by 7.04% in the post-introduction period as compared to the pre-introduction period and suggests that the antibiotic chart may have contributed to the reduced proportion of antibiotic prescribing. It is common for in-patients to receive one or more antibiotics subject to clinical considerations. Benefits such as broadened antimicrobial cover, and synergistic bactericidal or bacteriostatic effects, may warrant the use of combination antibiotics [30]. However, unnecessary concurrent antibiotic use, duplicate antibiotic cover or constant and unnecessary changes to prescribed antibiotics are common practices in the hospital setting [31]. This study recorded a mean ± SD number of antibiotics prescribed per patient in the pre-introduction period of 1.91 ± 1.14, and 1.96 ± 1.08 in the post-introduction period with no statistically significant difference (p=0.3679) across both study periods. A varying average number of prescribed antibiotics per in-patient admission has been reported, ranging from 1.29 to 2.1 [22,24,26,32,33]. A related study that reviewed a pharmacist-led AMS intervention reported a statistically significant difference in the mean number of antibiotics prescribed per patient during the intervention when compared to the period following its cessation (p=0.001) [34]. Thus, a clinical pharmacist may be of benefit in ensuring optimal antibiotic use using AMS strategies in the hospital setting. The most commonly used antibiotics were similar in both study periods and included ampicillin, gentamycin, and amoxicillin/clavulanic acid.

This is similar to reports from studies conducted in paediatric wards in South African hospitals [35,36] and an Ethiopian referral hospital [22] where ampicillin and gentamycin were reported to be the most frequently prescribed antibiotics. Concurrent administration of gentamycin and ampicillin in the empiric treatment of neonatal sepsis and severe pneumonia is recommended in the paediatric hospital-level Standard Treatment Guidelines (STG) in South Africa [37]. This may account for the frequent use of ampicillin and gentamycin, as cases of sepsis and pneumonia were predominant in both study periods. In addition, the duration of therapy increased by 0.55 days in the post-introduction period compared to the pre-introduction period. This could be explained by the 2.7-fold increase in the cases of sepsis as compared to the pre-introduction period. In addition, the paediatric hospital-level STG in South Africa recommends the treatment of neonatal sepsis for a duration of 10 days [37]. The duration of antibiotic therapy is often guided by clinical response and prolonged in bloodstream infections [38]. The appropriate route of administration for any medication should deliver the quantity necessary to produce the desired result without an adverse outcome [39]. Overuse of parenteral medicines constitutes irrational use of medicines [40]. This, alongside the challenges of medicine administration, discomfort to patients, increased cost, risk of infection and longer hospital stays make the oral route a preferred choice over the parenteral route [39]. The study ward frequently employed parenteral antibiotics in both study periods. The repeated use of parenteral antibiotics in this study may be associated with limited antibiotic choices in the hospital formulary, severity of the illness, unavailability of an alternate dosage form of the appropriate antibiotic as well as inadequate antibiotic stock on hand. Despite the possible adverse effects of the parenteral route of administration, such as the risk of infection, parenteral antibiotics have been documented to be the major route of antibiotic administration in several studies [22,23,26,41,42].

**Culture and sensitivity testing:** the need for appropriate culture and sensitivity testing in antibiotic selection cannot be overemphasized. It is essential that samples are appropriately and promptly obtained for testing and requested before initiating antibiotic therapy to optimize an accurate diagnosis [43]. The proportion of culture and sensitivity tests requested in the post-introduction period increased by 5.64% (from 77.98% in the pre-introduction period to 83.62% in the post-introduction period), although the difference was not statistically significant (p=0.710). In this study, cultures were requested more often than reported in a paediatric ward of a public hospital in South Africa, where laboratory tests were not requested in 69.7% of cases [35]. In addition to the above findings, the use of the antibiotic prescription chart in the post-introduction period was also reviewed. Successful implementation of an antibiotic prescription chart may improve compliance with appropriate usage and prescribing of antibiotics. The chart encourages clinicians to record the indication (prophylactic, empiric, definitive), dosage, duration and frequency of administration, allergy and abnormal renal or hepatic function [44]. Adherence to the documentation of clinical indicators on the antibiotic prescription chart is necessary to improve the review of antibiotic therapy using this AMS strategy [45]. The most omitted fields in this study included age (100%), a record of renal function (GFR/CrCl) (97.46%), time of antibiotic prescribing (83.62%), a record of culture and sensitivity results (80.17%), the rationale for antibiotic prescribing (50%) and record of infection source (50%). A study that audited an antibiotic prescription chart reported that commonly omitted fields included weight (45%), estimated glomerular filtration rate (36%) and allergies (32%) [20]. Antibiotics are frequently administered per kilogram body weight in paediatric patients, and this could account for the consistent rate of documentation of weight values on the antibiotic prescription chart. A probable reason for the inconsistent documentation observed could be the inconvenient and time-consuming process of completing the chart for clinicians as they may have several patients to attend to within a limited time frame. A computerized data entry system may increase the use and convenience of the antibiotic prescription chart [13]. There is, however, the potential for improvement in documentation of information on the antibiotic prescription chart as several fields were left incomplete. Prescribers are to be made aware of the need to complete all fields contained in the antibiotic prescription chart as this will assist in appropriate antibiotic selection and use. The commitment of clinical staff also plays an important role in achieving consistent and optimum benefits from the use of the antibiotic prescription chart.

**Limitations:** in the study ward, adherence to the antibiotic chart was insufficient, and as such, this study may not have entirely reflected the true picture of the public health sector based on the data collected. Another limitation of this study was that patient comorbid, chronic and immunosuppressed conditions were not linked to antibiotic prescribing, including prophylaxis with sulfamethoxazole/trimethoprim in patients with HIV. This may have influenced research findings such as the duration of antibiotic therapy and number of antibiotics prescribed.

## Conclusion

The outcome of this study indicates a statistically significant decrease in the proportion of patients prescribed antibiotics as well as a non-significant increase in the culture and sensitivity tests requested after the introduction of the antibiotic prescription chart. Evidence for proper implementation of the antibiotic prescription chart in this study is lacking. The antibiotic prescription chart is not being used to its full potential with poor documentation practices evident. Emphasizing the benefit of the antibiotic prescription chart as well as a regular review of antibiotic therapy using the antibiotic prescription chart by a clinical pharmacist and infectious disease physician may ensure optimal antibiotic use.

### What is known about this topic

- High rate of antibiotic prescribing;
- Surveillance studies regarding AMS practices and the impact of introducing an antibiotic prescription chart are scarce.

### What this study adds

- An antibiotic prescription chart can positively impact optimal antibiotic use;
- There is a gap in the effective implementation of the antibiotic prescription chart.
